# Initial Validity Evidence for the Student Health Equity Survey: Assessing Knowledge, Attitudes, and Capacity across Health Professions Programs

**DOI:** 10.12688/mep.21128.1

**Published:** 2025-11-07

**Authors:** Maranda C. Ward, Trudy Mallinson, Charles Cobbs IV, Rohini Ganjoo

**Affiliations:** 1 Department of Clinical Research and Leadership, The George Washington University School of Medicine and Health Sciences, Washington, District of Columbia, 20037, USA; 2 Department of Medicine, The George Washington University School of Medicine and Health Sciences, Washington, District of Columbia, 20037, USA; 3 Department of Biomedical Laboratory Sciences, The George Washington University School of Medicine and Health Sciences, Washington, District of Columbia, USA

**Keywords:** Health equity, health professionals, instrument development, competency-based education

## Abstract

**Construct:**

Health equity can be understood as an opportunity to achieve one’s fullest health potential and health professionals play a central role in advancing this aim. Thus, equipping health professional students with key competencies is paramount to achieving health equity.

**Background:**

There is no universally accepted definition of health equity. As such, standardized training of health professions students to address the unfair burden of poor outcomes among socially disadvantaged populations does not exist. These ongoing threats to health equity can be mitigated with competency-based training. We assess the health professions training programs at an academic medical center that has documented health equity values and no standardized or measurable health equity curriculum.

**Approach:**

We collected validity evidence for an instrument, the Student Health Equity Survey, to assess what students across nine health professions programs know and perceive about health equity concepts. Our 36-item instrument reflects the tenets of the Health Equity Framework. Content validity was assessed using an expert panel of faculty, staff and potential employers while content validity was further assessed alongside response process validity through cognitive testing-based focus groups. We used a Rasch analysis to assess for preliminary internal structural validity and construct validity. We strengthened our assessment of construct validity with an assessment of relationships between subscales through a series of cross plots and corresponding correlation coefficients for student measures on each subscale.

**Findings:**

Content and response process validity were established. Construct validity for the capacity, perspective taking, and reflection subscales included Cronbach’s alphas of 0.97, 0.71, and 0.82, respectively. The Cronbach alpha for the knowledge scale was low. Threats to construct validity are thought to be due in large part to sample selection bias.

**Conclusion:**

We developed a competency-based instrument to measure health equity knowledge, attitudes, and capacity of entering health professions students. Further research is necessary to test this instrument among health professions graduates entering the workforce.

## Introduction

One of the health goals of the United States is to achieve health equity ^1^. To assess progress towards achieving health equity, we measure the presence or absence of health disparities in the population that are shaped by uneven access to the social determinants of health (SDOH) ^2^. Since the impact of housing, healthcare, community safety, food and education on the health of populations are not discipline specific, these concepts need to be uniformly taught to current health professions students. As such, there remains a pervasive need to robustly educate students and trainees regarding health disparities and the SDOH. Yet, across the nation, health professions education differs within and across their curricula in such training. Additionally, students and trainees often learn about social disadvantage, racial bias, and systems of oppression through sporadic course electives, not as part of the required core curriculum ^3^. Even among educational programs that integrate health disparities into the core curriculum, there remains no defining framework or comparable assessment methods across curriculum efforts ^4^. In response, we designed a cross-disciplinary study within an academic medical center to operationalize health equity and standardize health equity competencies.

Health professions education reform is long overdue. The Commission on Education of Health Professionals for the 21 ^st^ Century identified in their report how pervasive health disparities necessitate curriculum reform within existing postsecondary education across health professions fields ^3^. In response, the National Academies of Science, Engineering, and Medicine identified teaching the SDOH to medical students as essential to combatting the underlying inequities in healthcare ^4^. The National Academies recognized the training gap in the existing curriculum and published a SDOH framework specifically for health professions education ^4^. Similarly, the GW Fitzhugh Mullan Health Workforce Institute is spearheading a Social Mission Metrics Initiative (SMMI) across 700 dental, medical, and nursing programs to create a system of validated metrics on how health professions training programs align to their social mission, with curriculum being one of 18 areas ^5^. The SMMI awaits validated metrics on curricular content related to health disparities and SDOH. Yet, without validated tools to develop robust health equity curriculum, health professions schools have experienced difficulty translating the SDOH framework into measurable learning outcomes ^6, 7^.

While health professions programs recognize the need for health equity curricula, there is limited literature measuring the impact of health equity training within health professions curriculum ^4^. The Liaison Committee on Medical Education (LCME) accreditation tool contains a section designated for medical institutions to report the integration of cultural competence and health care disparities in their curriculum ^8^. LCME defines this as “the faculty of a medical school ensuring that the medical curriculum provides opportunities for medical students to learn to recognize and appropriately address biases in themselves, others, and in the health care delivery process” ^8^. Despite being a key criteria required for medical schools to maintain accreditation status, a report of health disparities curriculum of LCME accredited medical schools during the 2017–2018 year, revealed that only 51 schools out of 151 (40.2%) documented racial disparity in their programs ^9^. This report underscored the urgent need for sustained curriculum and clinical-based training focused on implicit bias ^9^. Equally important is ensuring that curricular tools and outcomes measure requisite skills to effectively engage in this work ^10^. In general, existing learning traditions focus on knowledge and skill transfer, often neglecting the development of competencies like SDOH knowledge in the curriculum.

The Accreditation Council for Graduate Medical Education (ACGME) also requires competency-based training in health disparities; however, a study by Dupras and colleagues found few programs provided measurable educational curriculum in health disparities or quality control in such measurement ^11^. Unfortunately, existing literature regarding the inclusion of health disparities coursework shows that the competencies are covered in elective courses offered sporadically with no validated means to measure competency ^6, 7^. In addition, assessments of these programs rely largely on qualitative data such as self-assessments of attitudes towards marginalized communities lacking quantitative data such as skills/knowledge acquisition or long-term impact in the community ^6, 7^. For example, Reutter and Kushner emphasize the need to strengthen health inequity knowledge and its effects, as understanding population health enables clinicians to provide sensitive, non-judgmental care ^12^. Thus, a competency-based education offers a surefire method for health professions educators to equip their trainees to address health disparities.

For the aforementioned reasons, this research study includes a collaboration between several health professions’ training programs to operationalize the health equity concepts intended to translate into program competencies. We are invested in measuring our students’ health equity attitudes and capacity to determine how best to support them. The inadequate training regarding the knowledge of health equity among health professions students and trainees are exacerbated by the absence of a measurable curriculum designed to prepare them to address the issues that perpetuate health disparities.

The first aim of this study described here is to collect initial validity evidence on the health equity knowledge, attitudes, and capacity of incoming first-year health professions students across nine programs. We developed an instrument we call the Student Health Equity Survey (SHES) that measures incoming students’ training needs and educational support required through didactic, clinical, experiential, and co-curricular experiences. Our next aim is ongoing and focused on adding to the literature results from the SHES instrument that can assess health equity knowledge, attitudes, and capacity of newly enrolled health profession students. Our study will inform local curriculum reform and co-curricular programming efforts; conduct the first cross-disciplinary study within an academic medical center on how to uniformly measure health equity competencies; and serve as a model for how to conduct a mission-driven study in an academic medical center.

With evidence-based uniformity on how to measure health equity knowledge, attitudes, and capacity of health professions students, students will be able to rely on a foundational understanding of the psychosocial and geopolitical factors determining health outcomes of their patient populations. We believe students of training programs with measurable health equity-based competencies can better navigate perceived constraints within complex healthcare, government, and research systems.

### Survey development

The overall development of the SHES is based on published constructs describing the Health Equity Framework (HEF) and focuses on three foundational aspects: equality at the core of health outcomes, interacting spheres of influence, and life-course perspectives ^13^. Health equity competencies covered in this instrument are adapted from the Public Health Association of British Columbia and includes: health principles, policy, structure, critical thinking and analysis, communication, capacity and support, diversity, equity, inclusion and justice-based values, collaboration and advocacy (See Table 1) ^14^. Based on the content of these competencies, we divided the SHES into 6 subscales:

1.    assessing knowledge, parts I-II,

2.    translating knowledge into practice,

3.    assessing attitudes and beliefs,

4.    assessing perspective taking, (adapted from Scale of Ethnocultural Empathy)

5.    assessing stance, (adapted from the Multidimensional Approach to Individual Responses to Empathy scale);

6.    being reflective.

**Table 1.** Theory and competency development of the Student Health Equity Survey (SHES).

Health Equity Framework Tenet	Competencies	Instrument Subscales
Equity at the Core of Health Outcomes: agency, access, opportunity	Public Health Principles	Assessing Knowledge, 1 (Q1–3)
	Critical Thinking & Analysis	Assessing Knowledge, II (Q4–13)
	Communication	Translating Knowledge into Practice (Q14)
	Capacity and Support	Translating Knowledge into Practice (Q15–18)
Interacting Spheres of Influence: systems of power, relationships, individual factors, physiological pathways	Policy and Structure	Assessing Knowledge, II (Q4–13)
		Assessing Attitudes/Beliefs (Q19–23)
		Being Reflective (Q35–36)
	Diversity, Equity Inclusion, Justice-based Values	Assessing Perspective Taking (Q24–29) Being Reflective (Q33)
Life-Course Perspective: cumulative experiences across the lifespan	Collaboration and Advocacy	Assessing Stance (Q30–31) Being Reflective (Q32,34)
	Capacity and Support	Translating Knowledge into Practice (Q15–18)

We relied on items from the Scale of Ethnocultural Empathy for our SHES to assess perspective-taking ^15^. This original scale includes 4 subscales on empathic feeling and expression (15 items), empathic perspective taking (7 items), acceptance of cultural differences (5 itmes) and empathic awareness (4 items). We relied on 4 of the 5 subscales and selected 6 items: 2 items on empathic feeling and expression, 2 items on empathic perspective taking, and 2 items on empathic awareness. To assess student’s stance taking, we adapted items from the Multidimensional Approach to Individual Responses to Empathy ^16^. This scale includes multiple subscales: fantasy (9 itmes), perspective-taking (9 items), empathic concern (15 items), and personal distress (13 items). Of the 45-items on this published scale, the PIs selected two items that assess interpersonal reactivity from the perspective taking subscale. We edited one of the items from this published scale, ‘ *If I'm sure I'm right about something, I don't waste much time listening to other people's argument*s’. Students suggested that we replace the term ‘ *to other people’s*’ with ‘ *my peers’*. They clarified that the audience mattered for them. All of the other subscales were designed by our team.

Each of these subscales represent specific competencies (see Table 1). Survey answers were based on four-point Likert scale responses ranging from strongly agree to strongly disagree. We decided to change all the response options to a 4-point agreement response for consistency across scales (and simplicity for respondents). Finally, demographic questions were added to assess participant race, age, income, and program groups.

## Materials and methods

### Settings and participants

The SHES consists of 36 items to measure health equity knowledge, attitudes and capacity in incoming first year part-time and full-time certificate, undergraduate, post baccalaureate, graduate, and doctoral students from the School of Nursing and the School of Medicine and Health Sciences at an urban private academic medical center. The study was approved by the University’s Institutional Review Board (#NCR202535) for human subject protection where written consent was waived given the minimal risk and its impracticality to participate in cognitive and pilot testing of this instrument.

### Stages of survey validation process

**Figure 1.**
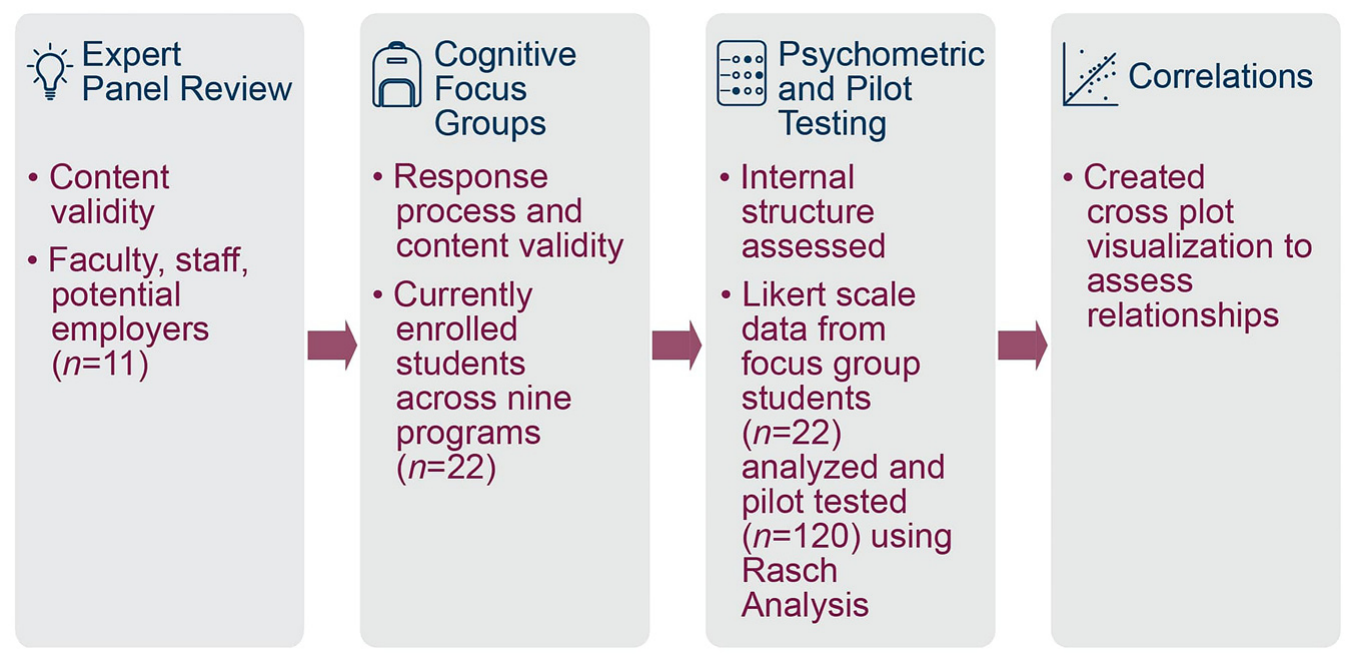
Stepwise process of collecting validity evidence for the Student Health Equity Survey (SHES).

The collection of validity evidence for the SHES scores involved four steps shown in Figure 1:

1) Content: convening an expert panel of faculty, staff, and potential employers ( *n*=11);

2) Response process and content: conducting cognitive ‘think-aloud’ testing using focus groups of currently enrolled students ( *n*=22);

3) Internal structure: initial testing of Likert scale data collected from the focus group students ( *n*=22) using Rasch analysis and pilot testing of the updated tool with students ( *n*=120) using Rasch analysis; and

4) Correlations: creating cross plot data visualizations to examine expected relationships.

### Content

To collect validity evidence based on the content of the SHES items, we convened an 11-member expert panel. Initially, we identified 3 faculty members with a background in public health, health disparities, public policy and/or community-engaged research who could help us determine the health equity concepts, values and skills required of students. Using a snowball sampling approach, they further identified an additional 8 members including other faculty, staff, and potential employers. The experts reviewed the study aims and provided input on the SHES items that were then incorporated into the tool.

### Response process and content

To collect both response process and additional content validity evidence, we enrolled students for a phase of cognitive testing of the SHES items. Recruitment materials, including fliers and email announcements were shared with program directors of each collaborating program to distribute via student listservs and course learning management systems. Student participation was voluntary and anonymous and those who consented and enrolled (n= 22) received an online Qualtrics link to complete the SHES, followed by enrollment in a focus-group. The two co-PIs conducted five small cognitive focus groups consisting of four - six participants each, lasting about 90 minutes and audio recorded via videoconferencing. Video cameras of participants were turned off during the focus group to protect anonymity. The recordings were then reviewed for emerging themes. These cognitive focus groups focused on usability of the survey platform as well as the order, perception and interpretation of each item to ensure that wording and instructions were understood ^5, 6^. We asked students to describe how they arrived at their responses rather than their actual responses to the items.

### Internal structure

Using a Rasch analysis, we assessed the internal structure of the SHES scores (n=22). The Rasch analysis is an iterative process to maximize measurement precision and was conducted using WINSTEPS software to apply a rating scale model (WINSTEPS®: Rasch Measurement [computer program]. Version 5.1.7.0 2023. We ran the Rasch analysis to measure the evaluation of rating scale category structure, dimensionality, and measurement precision.

**Rating Scale Category Structure.** The 13 multiple-choice items on the *Knowledge* subscale of the SHES were coded on a 4-point Likert scale ranging from strongly agree and agree to disagree and strongly disagree. We re-coded them as correct (agree and strongly agree) and as incorrect (disagree and strongly disagree). We then compared the *Knowledge* subscale item responses to the scoring key and assigned correct responses a value of 1, and incorrect responses a value of 0. Higher scores on this knowledge subscale represented greater student knowledge of health equity.

In general, as seen in Table 2, stable category calibrations require a minimum of 10 responses per category. Less than 10 responses were seen for items 2, 6, 10, and 11 in the *Knowledge* subscale. Category 1 (strongly disagree) was under-used (<10 responses) or not used at all for all items of the *Translating Knowledge to Practice, Perspective-taking, and Reflection* subscales. Category 2 (disagree) was under-utilized for items 1, 2, 6, 7, 10, 11, and 13 of the *Stance* subscale and all items except #4 of the *Reflection* subscale. All categories were arranged monotonically (i.e., in order from low to high) as expected, however low utilization of the strongly disagree and disagree category resulted in relatively wide standard error indicating a lack of precision.

**Table 2.** Item difficulty calibrations, Standard Error, Infit Mean square residuals, and Standard scores from the pilot psychometric testing of the updated tool with students ( *n*=120) per Rasch analyses. Standard error: SE; Infit mean square residuals: MnSq; Standard scores: Zstd. *Denotes statistical significance. ^Denotes items changed due to feedback during the iterative process.

Item Text	Item Calibration	SE	MnSq	Zstd
**Knowledge Subscale (n= 13 items)**				
^1. Eliminating individual risk behaviors (i.e. smoking, poor eating habits etc) will reduce health disparities.	4.25	0.33	0.85	-0.64
3. Health equity focuses on improving the health of minority populations.	3.78	0.29	1.30	1.57
7. If the U.S. spends more money on healthcare, it will automatically improve life expectancy.	1.90	0.21	1.19	2.16
^8. Health professional shortages contribute to the high rates of U.S. maternal mortality.	0.59	0.22	1.10	0.99
^9. Eating healthy is an issue of personal responsibility, not an issue of access.	0.00	0.24	0.76	-1.97
4. Social policy is health policy.	-0.19	0.25	0.98	-0.08
5. Where people live determines how long they live.	-0.62	0.28	0.86	-0.79
^12. The health disparities that LGBTQIA+ populations face is underreported due to the lack of standardized sexual orientation and gender identity data.	-0.68	0.29	0.89	-0.55
13. Chronic stress and poor diet can change the expression of genes.	-0.70	0.29	1.02	0.15
2. Improving the health of the least socially advantaged groups improves the health of all groups.	-1.43	0.36	1.00	0.10
10. Health literacy in the U.S. is only an issue for English language learners.	-1.72	0.40	0.93	-0.11
6. Access to healthcare is more than having insurance.	-2.10	0.47	0.90	-0.14
^11. Health disparities affect populations across the lifespan.	-3.08	0.72	1.01	0.23
**Translating Knowledge into Practice Subscale (n = 7 items)**				
^6. I have the capacity (i.e. time, ability, support, resources) to redistribute power in my current professional role to advance health equity.	0.73	0.23	1.00	0.05
^5. I have the capacity (i.e. time, ability, support, resources) to redistribute power in my current student role to advance health equity.	0.69	0.18	0.95	-0.33
7.I feel supported in my current role to use policy and/or practices to address health disparities.	0.51	0.19	1.33	2.21
^3. I feel confident in my ability to translate my health equity understanding into current practice via teaching, service, research, and/or patient care.	-0.13	0.23	0.61	-2.64
1. I understand how to explain the concept of health equity to other people.	-0.29	0.19	0.77	-1.80
4. I feel confident in my ability to translate my health equity understanding into future practice via teaching, service, research, and/or patient care.	-0.68	0.19	0.62	-3.19
2. I feel comfortable talking about the impact of racism on health outcomes.	-0.83	0.19	*1.53	3.40
**Perspective taking (n=11 items) and Stance Subscales (n = 2 items)**				
3. It is good practice to use race as a risk factor when interpreting population health data.	2.46	0.13	*1.73	5.23
5. Expecting racial and ethnic minority populations to change cultural behaviors to improve their health outcomes is insensitive.	0.71	0.14	0.99	-0.06
9. It is difficult for me to relate to stories in which people talk about racial or ethnic discrimination they experience in their day to day lives.	0.6	0.14	1.22	1.63
8. It is difficult for me to put myself in the shoes of someone who is racially and/or ethnically different from me (R).	0.34	0.14	1.02	0.23
12. If I’m sure I’m right about something, I don’t waste much time listening to my peer’s arguments.	0.34	0.14	1.15	1.12
^2. Historical trauma (i.e. slavery, trail of tears) only impacts previous generations of U.S. minorities.	-0.16	0.15	1.09	0.70
6. When other people struggle with racial or ethnic oppression, I share their frustration.	-0.19	0.15	0.59	-3.58
4. When research does not include racially diverse samples, the treatment modalities and clinical standards are biased towards White middle-aged men.	-0.48	0.16	1.09	0.71
10. I can see how racial or ethnic groups are systematically oppressed in our society.	-0.51	0.16	0.96	-0.29
^13. I believe that there are two sides to every question and try to look at them both when talking with peers.	-0.53	0.16	1.06	0.51
7. When I interact with people from other racial or ethnic backgrounds, I show my appreciation of their cultural norms.	-0.59	0.16	0.57	-3.78
11. I am aware of how society differentially treats racial or ethnic groups other than my own.	-0.84	0.17	0.74	-2.11
1. Being racially discriminated against impacts one’s health.	-1.15	0.18	0.84	-1.14
**Reflection Subscale (n=5)**				
4. Having racially and ethnically diverse faculty, administrators and peers affect the distribution of power in my profession.	0.67	0.19	1.23	1.36
2. I believe it is important to reflect on my own privilege for how I benefit from social structures that may oppress other groups.	0.16	0.2	1.11	0.66
3. I believe that my health professions training program has a role to play in either fighting against or maintaining health inequities.	-0.08	0.2	0.83	-1.01
5. Health professionals must consider the impact of their social status for how it shapes community relationships.	-0.14	0.21	0.73	-1.67
1. I have a professional responsibility to work towards social change.	-0.61	0.21	1.00	0.05

**Dimensionality.** Preliminary results indicated that *Perspective-taking* and *Stance* subscale items were best combined into one *Perspective-taking* subscale and *Capacity* and *Translating Knowledge to Practice* items were best combined to form a *Translating Knowledge to Practice* subscale (preliminary results not presented). In both cases, combining these groups of items improved coefficient alpha and item fit statistics. Going forward for the pilot test, we surveyed students using these 4 subscales (rather than the initial six) and we only report the results for these 4 subscales.

**Measurement Precision.** Person separation reliability (PSR) is an indication of measurement precision across all items and is interpreted in a similar manner as Cronbach's alpha. Values >0.70 are designed for group level use and >0.90 for individual level use. Our initial analysis of all items combined resulted in a PSR 0.83. However, we interpreted this value as being artificially inflated due to the presence of multiple dimensions within subsets of the items ^16, 17^. The principal component analysis of residuals (PCAR) suggested multiple dimensions within the items (variance explained by the measures 37%, eigenvalue 5.0 and variance explained in the first contrast 13%,). Factor loadings indicated that the items on the *Translating Knowledge to Practice* subscale were distinct from the *Perspective Taking* and *Reflection* subscale items (disattenuated correlation of 0.40.) and from *Knowledge* subscale items (disattenuated correlation of 0.68). Nine of the 36 items misfit (infit mean square <0.7 or >1.3 and standardized Z >2.0).

### Correlations

Upon completion of the response process and content analysis, we conducted a pilot test with enrolled students in the following semester. Students who had already participated in the earlier steps were not included. We sent an email announcement and flier with a QR code linked to the survey to program directors to share with students and course instructors about the opportunity to participate in this final process of testing the SHES items. Two additional reminders were sent to encourage students to participate in the study during the pilot period. We utilized the Rasch analysis to measure the student responses (n=120) for evaluation of dimensionality, measurement precision, and to construct cross plots. 

### Analytic framework

We relied on Kane’s framework for a portion of the SHES subscales ^18^. For instance, the *Knowledge* and *Perspective-taking* items on the SHES have ‘correct’ responses that allowed us to generate a score ^16^. For this reason, our interpretation-use argument was that the higher the score for health professions students, the more competent they are in health equity. This argument does not apply to the subjective scales on *Translating Knowledge into Practice* or *Reflection* as these subscale items focus on one’s opportunities to demonstrate learning in real-time and exposure to diverse communities. 

Our interpretation-use argument is based on several assumptions. First, a high score on *Knowledge* and *Perspective-taking* scales means that health professions students understand the social determinants and can describe the impact of social conditions on population health. Second, our assessment tool, the SHES, is consistent. Third, we believe that program directors understand how to interpret the SHES and that scores are reproducible. Finally, we believe that the scores would relate to real-world experiences in professional and clinical settings.

## Results

### Content

We added clarifying language to the SHES based on comments from the expert panel reviewers. Some comments were separating an item into two parts for clarity, use of definitive wording like ‘determine’ instead of ‘influence’ and ‘spend time’ instead of ‘waste’. Before moving on to the next step, we ensured that the expert panel approved the final wording and sequence of all the items.

### Response process and content

We utilized a pragmatic, iterative approach while conducting the focus groups by reviewing feedback from each group and making concurrent changes to the SHES before distribution to the next group of students. This was done to ensure that the next group of students would weigh in on the changes that were made. The thematic analysis from the focus groups helped inform the revision of the SHES. Key findings from the focus groups were that students were in support of adding an additional response category of ‘not yet practicing’ to the Likert scale for two items that assessed current professional role/s.

We revised the text of 6 items based on these cognitive testing-based focus groups and indicate the revised items by “^” in Table 2. The most common changes made included: clarifying wording (e.g., changing ‘cause’ to ‘contribute’, ‘eliminate’ to ‘reduce’, ‘people’ to ‘my peers’), item reordering to keep like content together, adding additional content, providing parenthetical examples to enhance clarity (e.g., “individual risk behavior”), and separating items regarding knowledge translation into current and future roles. Below are examples of some edits made:

Original Item: ‘ *Health professional shortages cause the high rates of U.S. maternal mortality’.*

New Item: ‘ *Health professional shortages **contribute** **to** the high rates of U.S. maternal mortality’.*

Original Item: ‘ *Eating healthy is not an issue of access, but of personal responsibility’.*

New Item: ‘ *Eating healthy **is** an issue of personal responsibility, **not** an issue of access’.*

When we asked if students believed any concepts or content were missing from the SHES, two key areas emerged across the focus groups. The first was about including a lifespan approach, which aligned with our theoretical framework. The second was specifically naming sexual and gender minoritized groups as an underrepresented population who experience growing disparities. So, we created draft items that were shared with the expert panel for wordsmithing and fine tuning resulting in the inclusion of two additional survey items at this stage of the validation process which included: ‘ *Health disparities affect populations across the lifespan’* and ‘ *The health disparities that LGBTQIA+ populations face is underreported due to the lack of standardized sexual orientation and gender identity data.’*

Given the range of student experiences, it was advised that we acknowledge that some students have not yet been employed and would not be able to respond solely to a question on their professional role. We edited the survey in two ways to address this omission. First, we added a new item specifically asking about one’s student role. Secondly, for the two items that assessed current (professional) roles, we added an additional response category to the Likert scale as ‘not yet practicing’. Students were adamantly opposed to having to rely solely on close-ended responses for any of three capacity-related items given how nuanced their responses were. For this reason, we added an open-ended field for them to explain their responses to the previous two items.

Original item: ‘ *I have the capacity to redistribute power and resources in my current professional role to advance health equity’.*

New item: ‘ *I have the capacity **(i.e., time, ability, support, resources)** to redistribute power and resources in my current professional role to advance health equity’.*

New item: ‘ *I have the capacity **(i.e., time, ability, support, resources)** to redistribute power and resources in my current **student** role to advance health equity’.*

New Item: ‘ *Feel free to explain your answer (in 17a-b)’*

Based on focus group comments, we provided additional options for the following demographic items: gender (e.g., ‘ *more than one gender’, ‘prefer not to respond*’ and ‘ *prefer to self-identif*y' (with a write in option), military status (e.g., *‘national guard*’, ‘ *reserve*’, ‘ *retired*’, and ‘ *military dependent*’), and financial situation (e.g., ‘ *allows me to love comfortably with loans or support of someone else*’ ‘ *finances just meets my needs with loans*’), and a write in option ‘ *if none of the above applies to you, please briefly explain her*e’).

### Internal structure

Based on the preliminary Rasch analysis (described above) we revised the rating scale category for the *Translating Knowledge subscale* and created 4 subscales instead of 6. This version was used for pilot testing with 120 students.

Participants were predominantly white (55%), female (72.5%), in the 23 - 30 age group (42%) and from the Biomedical Laboratory Sciences programs (76.7%) as seen in Table 3. One student did not share their age. Most were pursuing graduate-level certificates (43.3%) and 31.7% had bachelor’s degrees. Of the 121 students who started the SHES, 120 students completed it and were included in the Rasch analysis.

**Table 3.** Description of the demographic information of survey respondents in the preliminary structural testing (n=120).

Racial/Ethnic Group Identity	Total	Percent
American Indian/Alaskan Native	0	0
Asian/Pacific Islander	22	19.2
Black/African American	16	13.3
Caucasian/White	55	45.8
Hispanic/Latinx	16	13.3
Multiple	6	5
Other	5	0.8
**Sex**		
Female	87	72.5
Male	31	25.8
Other	2	1.7
**Age (n=119)**		
20–25	40	33.6
26–30	42	36.2
31–35	25	21.0
36–40	9	7.6
41–45	0	0.0
46–50	3	2.5
**Current Program of Study**		
Biomedical Laboratory Science	95	79
Clinical Research and Leadership	1	0.8
Medicine	12	10.1
PA/MPH Dual Degree	3	2.5
Physician Assistant	8	6.7
Undisclosed	1	0.8
**Highest Level of Education**		
High School	0	0
Associate Degree	18	15
Bachelor’s Degree	89	74.2
Master’s Degree	11	9.2
Doctorate Degree (MD, PhD, DPT)	1	0.8
Certificate	1	0.8
**Degree in Progress**		
Certificate	52	43.3
Bachelor’s Degree	39	32.5
Master’s Degree	14	11.6
Doctorate Degree (MD, PhD, DPT)	9	7.5
Dual Degree (PA/MPH)	2	1.7
Other	6	5
**Year in Program**		
Year 1	37	30
Year 2	35	29
Other	48	40
**Financial Status**		
Finances do not meet my needs	19	15.8
Finances just meets my needs	19	15.8
Finances meet my needs with a little left over	21	17.5
Finances allow me to live comfortably	15	12.5
Finances allow me to live comfortably (with loans or support of someone else)	14	11.7
Finances just meets my needs with loans	10	8.3
More than one applies	22	18.3

**Decision to report separate subscales.** Results of the subsequent pilot testing on 120 students, suggested that all four subscales captured distinct constructs and were thus reported individually. Specifically, PSR for each subscale was *Knowledge* 0.26, *Translating Knowledge into Practice* 0.72, *Perspective-taking* 0.63, *Reflection* 0.58. Only 5 items misfit (infit MnSq >1.3; ZSTD >2.0); and only 2 underfitting (MnSq >1.3) – Item 2 ( *Capacity* subscale) and Item 3 ( *Stance* subscale) ( Table 2). PCAR as demonstrated by Eigenvalues for each subscale ( *Knowledge* 1.97, *Translating Knowledge into Practice* 2.0, *Perspective-taking* 2.5, *Reflection* 1.9) indicated each subscale reflected discrete content.

**Person targeting for each of the subscales.** For all subscales, student average scores were indicated by logit values. When student scores and item calibrations were aligned, mean student values were within ±0.5 logits of the mean item difficulty. We found that the *Knowledge* subscale student mean was 1.58 SD 1.19, *Translating Knowledge to Practice* subscale student mean was 0.93 SD 1.75, *Perspective-taking* subscale student mean was 1.61 SD .91, and *Reflection* subscale student mean was 1.76 SD 1.57, indicating all mean student measures were well above mean item difficulties. Table 2 provides item calibrations and infit mean square (MnSq) presented in hierarchical order (high to low item calibrations). For each subscale, the ordering of the items aligns with expectations and calibration.

### Measuring reliability

Internal consistency was measured by Cronbach’s alpha and used to determine how well items within the given subscale fit together. Cronbach’s alpha values of 0.7 are considered adequate levels of reliability for a survey ^17^. While parts of this survey resulted in adequate scores, other items in the survey scored far lower than our desired value of 0.7. The lowest scoring subscale of the survey was the *Knowledge* (alpha = 0.46) subscale, with the 13 items designed to ascertain students’ knowledge about health equity, the causes of health disparities, and the role of health care professionals in promoting health equity. The remaining 3 sub scales had acceptable to high internal consistency: *Translating Knowledge into Practice* (alpha=.97), *Perspective-taking* (alpha=.71), and *Reflection* (alpha=.82).

The Cronbach’s alpha for the *Knowledge* subscale was likely low at 0.46 due to the similar range of knowledge detected by this survey with most respondents answering most items correctly. We believe that the sample population had high levels of knowledge being existing students in later years of study rather than entering students (only 30% of the population were in year 1 of their study) with no formal training. While the reliability is low, further analysis reveals that current students know what health equity is and the issues that lead to health disparities.

The *Translating Knowledge into Practice* subscale had the highest Cronbach’s alphas at 0.97 while the Cronbach’s alphas for the *Perspective-taking* subscale was 0.71. Both these subscales show uniformity of answers and clustering of data. One possible explanation is that the items are straight forward with obvious socially desirable answers. Yet, this may not be the case since many of the items in this section were taken directly from published data geared toward validating a scale used to assess ethnocultural empathy ^15^. These subscales were designed to test the respondent’s ability to empathize with those from other racial/ethnic groups. It has long been theorized that the U.S. white population, which has historically accounted for most healthcare providers, lacks an ability to empathize with other racial/ethnic groups due to limited interracial experiences ^17^. Of our respondents, 45.8% identified as Caucasian/white.

The *Reflections* subsections of the survey include items that assess the respondent’s capacity and comfort as a student and professional to translate their knowledge into practice with a Cronbach’s alpha of 0.82. Yet, most were unable to identify solutions or describe their role as professionals to promote health equity. This perspective may be further skewed as most respondents came from biomedical laboratory sciences, a field traditionally removed from providing direct patient care. These subscales capture a wide range of answers from respondents and reveal a common trend: most students expressed confidence in their current roles but lack uncertainty in their ability to translate their knowledge into future practice. Although these subscales scored well, it is notable that most of the respondents answered questions similarly. This may be due to the sample population of our pilot test including students with similar views being the ones who voluntarily participated.

### Correlations

We assessed relationships between subscales through a series of cross plots and corresponding correlation coefficients for student measures on each subscale. We examined the association between the subscales with negative values representing lower performance and positive values representing higher performance in the cross plots. The lower left region of each plot indicates respondents scoring lower on both measures; the upper right region indicates respondents scoring higher on both measures.

*Knowledge* vs *Translating Knowledge into Practice* ( Figure 2A). The responses reflected in Figure 2A depict a large number of respondents reporting high levels of knowledge and perceiving themselves as having substantial capacity to put their knowledge into practice (upper right region). About 20% of student measures indicated high knowledge but low capacity (lower right region). This suggests that there is still much room for improvement when it comes to providing students with the capacity to act on their knowledge. The correlation coefficients of r=.1153 reflects the relatively greater variability in students on the capacity sub-scale and the handful of those with average levels of knowledge but who report high levels of capacity to act (top middle region).

**Figure 2A.**
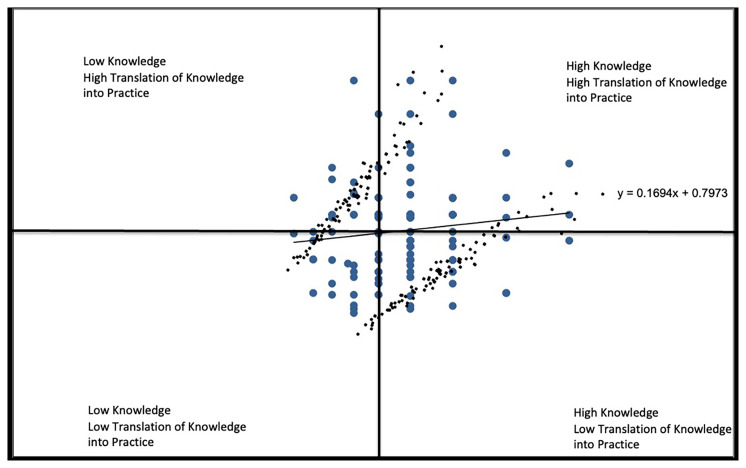
Knowledge vs. Translating Knowledge into Practice subscales.

*Knowledge* vs *Perspective-Taking* ( Figure 2B), the responses here indicate a positive relationship with students with less knowledge reporting a weaker stance on health equity and students with greater knowledge reporting a stronger stance on health equity as reflected in the correlation coefficient r=.3778. 

**Figure 2B.**
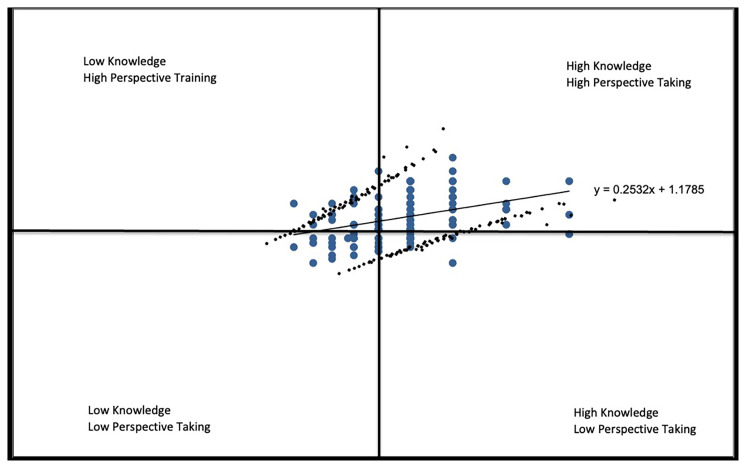
Knowledge vs. Perspective-taking subscale.

In *Knowledge* vs. *Reflection* ( Figure 2C), similarly, we found a positive relationship with students with less knowledge reporting less self-reflection and the majority of respondents, who had substantial knowledge, reporting high levels of self-reflection with a correlation coefficient of r=.4750.

**Figure 2C.**
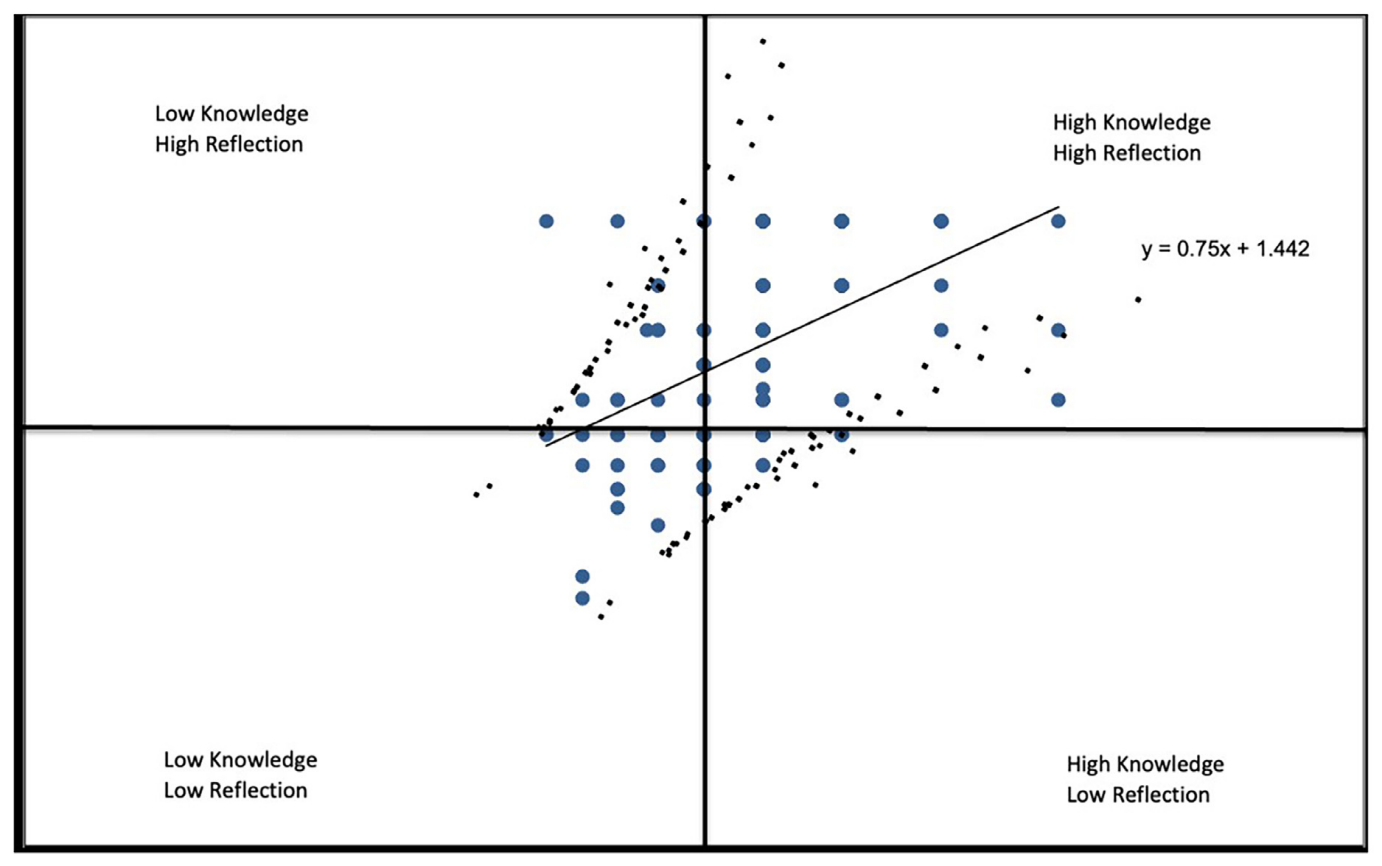
Knowledge vs. Reflection subscale.

For *Perspective-taking* vs. *Translating Knowledge into Practice* ( Figure 2D), the responses indicate nearly 30% of students express a strong stance on health equity but do not perceive themselves to have the capacity to put their beliefs into action (lower right region) with a correlation coefficient of r=.3764.

**Figure 2D.**
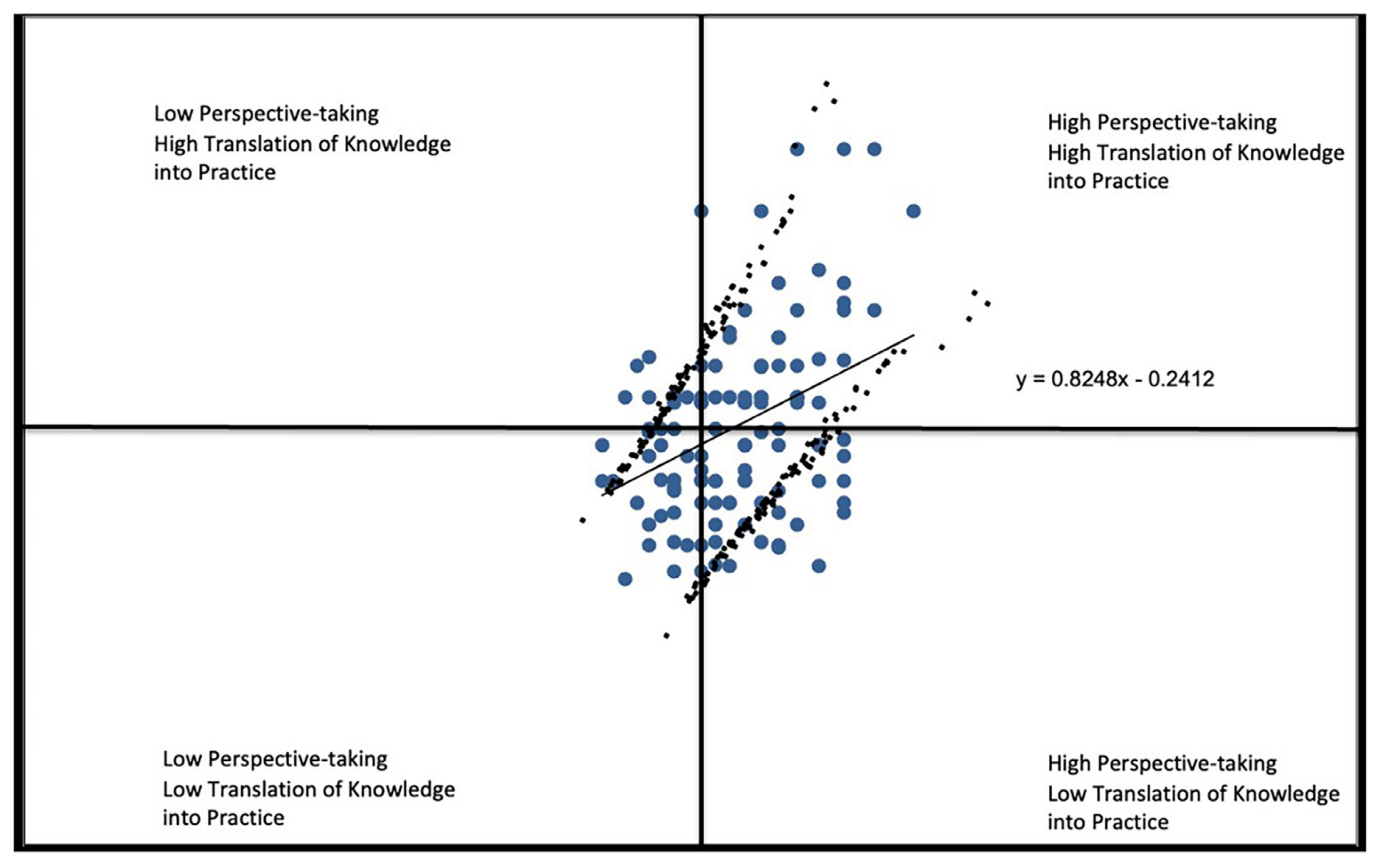
Perspective-taking vs. Translating Knowledge into Practice subscales.

In *Capacity* vs. *Reflection* ( Figure 2E), the responses indicated most respondents perceived high ability for self-reflection, although 24% of all respondents perceived themselves as having limited capacity to put the knowledge into practice with a correlation of coefficient r=.0072.

**Figure 2E.**
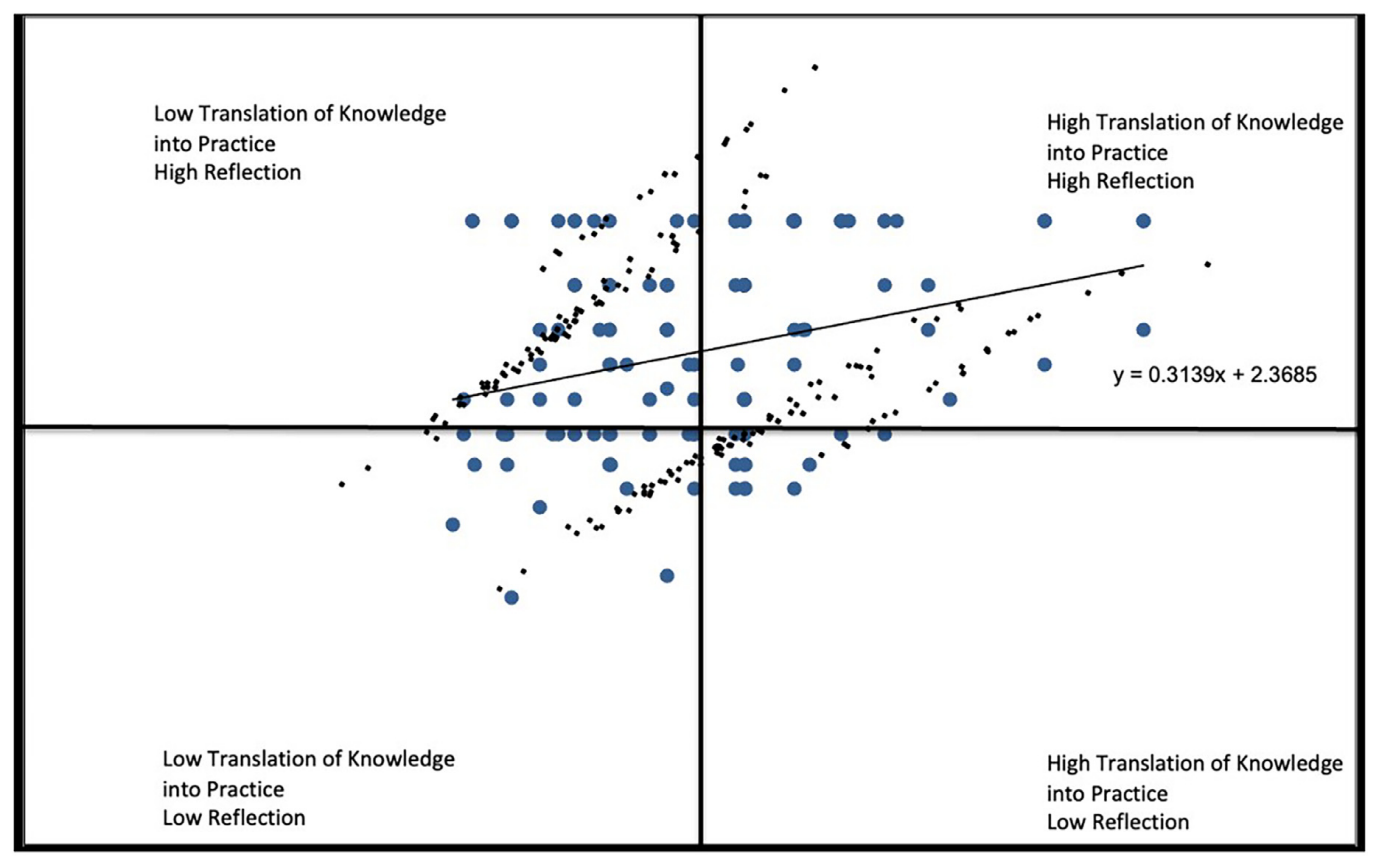
Translating Knowledge into Practice vs. Reflection subscale.

Finally, in *Perspective-taking* vs. *Reflection* ( Figure 2F), most respondents perceived themselves to have a high capacity for self-reflection and as taking a strong stance on issues of health equity reflected by the correlation coefficient of r=.6697.

**Figure 2F.**
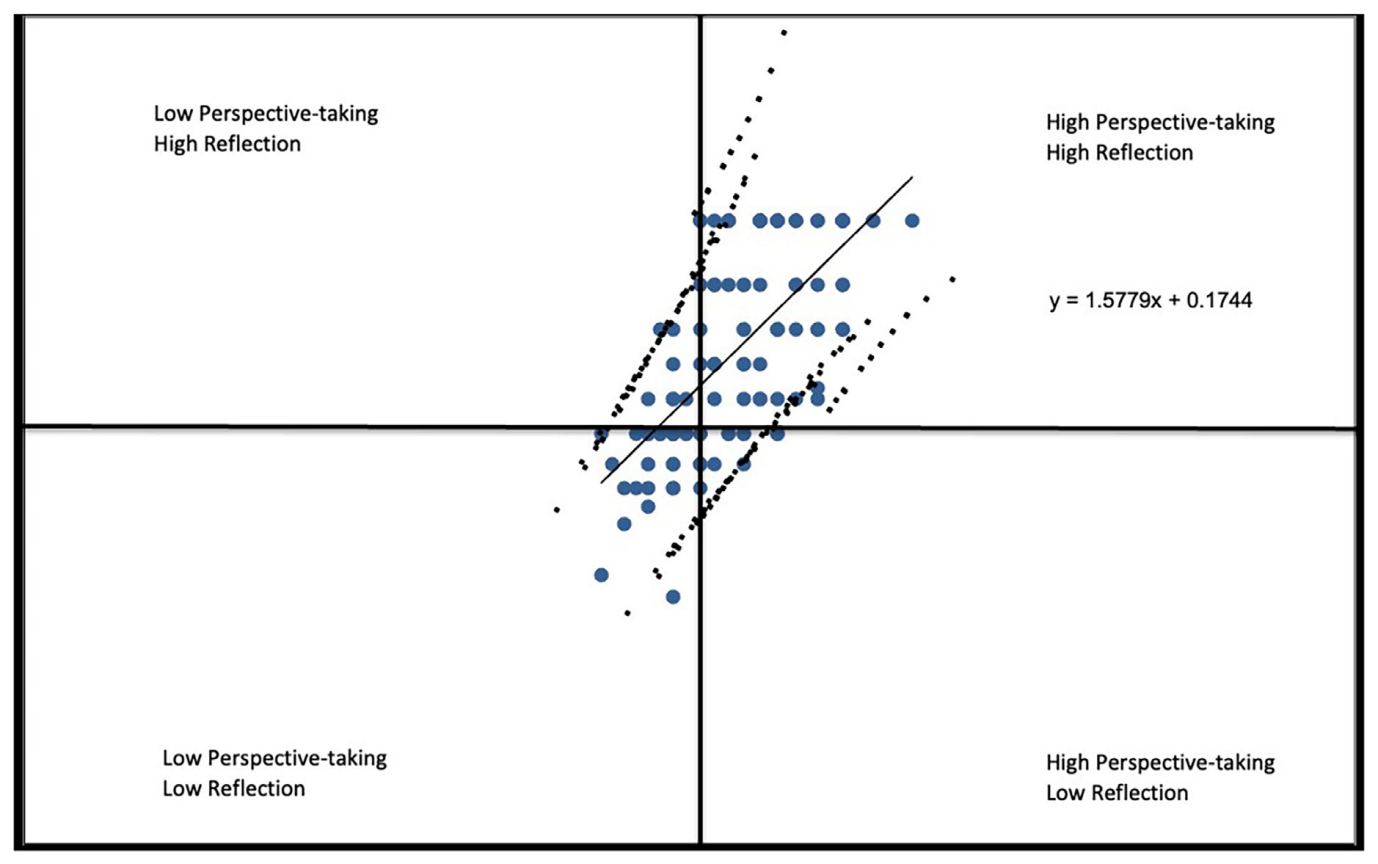
Perspective-taking vs. Reflection subscales.

## Discussion

The goal of our research is to offer a standardized set of health equity competencies that can be measured across health professions programs. We recognize that one of the greatest threats to this aim is that the concept of health equity is not universally understood ^17, 18^. Varied understandings of health equity leads to varied instruction and assessment of health equity content ^19^. According to a review of peer-reviewed and gray literature, health equity has been defined in three distinct ways: an idealistic attainment of health possibilities; a measurable absence of disparities; and/or a structural focus on opportunities for health ^2, 19, 20^. We agree with the prevailing literature that defines health equity “as the highest attainment of health for all populations'' ^2^. While we did not treat the “absence of health disparities” as a definition, we do rely on it as an important population health metric ^21^. We also believe that a “focus on opportunities for health” is not a definition but instead frames the set of health equity competencies necessary to eliminate structural inequities ^21^.

There remains great value in defining health equity in a standardized way across health professions programs so curricula can be designed to measure a set of key health equity competencies. For this reason, we selected the science and justice-based Health Equity Framework as a theoretical guide for the type and scope of knowledge, attitudes, and capacity necessary to address the social, cultural, and political population-level challenges to achieving health equity ^4^. In this way, the set of learner competencies we selected reflect what is widely accepted by the American Public Health Association and Association of American Medical Colleges about health equity but also measurable via our SHES instrument. By integrating SHES into health professions programs, institutions can standardize the measurement of health equity competencies, facilitating the comparison of outcomes across different cohorts and programs. This standardization can also support accreditation processes, as it provides reliable data on students' preparedness to address health disparities. Moreover, the SHES can inform continuous improvement efforts, guiding curriculum adjustments and the development of targeted interventions to enhance student learning outcomes.

In establishing a standardized and measurable health equity competency, we found distinct dimensions of competencies that align with the Health Equity Framework. For instance, our SHES is not a single scale but consists of unique subscales. While there is a relationship between the subscales, doing well in one content area does not translate into doing well on the others. It is clear that student responses varied in their competence on the subscales and our students in particular were least competent in the *Translating Knowledge into Practice* and *Reflection* subscales. Additionally, students who demonstrate ‘high knowledge’ did not translate into ‘high translation’ indicating that these are two separate and worthwhile competencies that need to be taught and assessed separately in health equity curriculum and training efforts.

Our findings on the relationships between health equity competencies, along with evidence supporting their validity are crucial for shaping health professions training. Health equity curriculum needs to be intentionally crafted to incorporate these competencies providing opportunities for learners to engage in practical application. Besides curriculum design, our research underscores the distinctiveness of health equity competencies, highlighting the intricate challenges of enhancing health equity knowledge and capacity across various training programs. Despite the scarcity of studies measuring and assessing health equity competencies among students and trainees, our study provides valuable insights that contribute to expanding the current understanding of the training the healthcare workforce. Our findings offer a critical foundation for conducting longitudinal studies in advancing health equity education and ensuring that future healthcare professionals are well equipped to address disparities in healthcare delivery. 

### Limitations

Our use of Rasch analysis, while a powerful tool, is not without its challenges. The method's sensitivity to different student types may introduce variability in the results ^22^. It is important to note that the validity evidence primarily reflects responses from students in later semesters of enrollment. While survey responses were obtained from a wide racial/ethnic group, there was an overwhelming response from students in one program with most (70%) respondents studying in their second year or beyond. In similar survey validation studies, participants were limited to first year students with desirable results ^10^. The SHES demographics we initially assessed such as sex was changed to gender identity in later versions and we included more options for race/ethnicity (See Appendix A). This study took place at the height of the COVID-19 pandemic in December 2020 and was, therefore, marked by a continuous need to update the timeline. The intricacies, time sensitive and iterative nature of these validation processes were compounded by the unprecedented challenges of working across multiple programs during a pandemic. Additionally, very high scores (alpha>0.90) may have suggested some item redundancy. Our squared correlations indicate the proportion of change in one measure accounted for by another measure was low for high level factors- which is not uncommon in educational research. Despite these challenges, we remained committed to producing reliable and insightful outcomes that could prove valuable for researchers in the field. We anticipate that our next steps would be to examine any differences in descriptive statistics across the correlations to potentially reveal any attenuation in the correlations due to group membership.

## Conclusion

With future health professionals poised to play a pivotal role in mitigating health disparities, it is important that educators and facilitators of health professional training programs measure students’ health equity-based competencies. Our analysis methods, processes, and interpretations may be of benefit to future researchers intending to validate or assess the latent structure of a new or modified psychometric measurement tool consisting of ordinal response sets. Our study findings are also useful for faculty and program administrators who determine program curriculum maps and report accreditation data who need standardized measures of health equity curriculum content and measurable competencies. To our knowledge, our study is the first to create a practical instrument to uniformly assess the health equity knowledge, attitudes and capacity of students enrolled in different health professions programs.

## Ethical approval statement

This study was approved by The George Washington University (GW) Institutional Review Board (IRB) #NCR202535.

## Figures and Tables

**Table:** 

Demographics
37. Age: Numeric Age
38. Sex (select one): F (female/f/she/her/hers/woman), M (male/cis-male). O (other)
39. Racial and Ethnic Identity (Select All That Apply): P (Asian/Pacific Islander), A (American Indian/ Alaskan Native), B (Black/African American), W (Caucasian/White), H (Hispanic/Latinx), O (Other), M (Multiple)
40. Military/Veteran Status: 1 (Active Duty), 2 (National Guard), 3 (Reserve), 4 (Veteran), 5 (Retired), 6 (Military Dependent), 7 (Non- military/Veteran)
41. Zip code of permanent residence (immediately before beginning your GW program):
42. Currently employed?: 1 (Yes), 2 (No)
43. Job title: 1 (Service Industry), 2 (Finances just meet my needs), 3 (Finances meet my needs with a little left), 4 (Finances allow me to live comfortably), 5 (Finances allow me to live comfortably (with loans or support from someone else)), 6 (Finances just meet my needs with loans), M (Multiple)
44a. What is your current ﬁnancial situation (i.e. loans, debt, other support)?
44b. If none of the above options in #44a applied to you, please brieﬂy explain here.
45. School: (select one)
46. Program of Study (select one): 1 (Physician Assistant), 2 (PA/MPH), 3 (MLS), 4 (Medicine), 5 (Other)
47. What is your highest level of completed education? (select one): 1 (High School Diploma), 2 (Associates), 3 (Dual Degree), 4 (Master’s), 5 (Doctorate (i.e. MD, PhD, DPT)), 6 (Other)
48. Current Degree Type (select one): 1 (Certificate), 2 (Bachelor’s), 3 (Dual Degree), 4 (Master’s), 5 (Doctorate (i.e. MD, PhD, DPT), 6 (Other)
49. Current Year in School (select one): YI (First), Y2 (Second), Y3 (Other)

## Data Availability

We agree to make freely available all data supporting the results and analyses in this paper. All data can be accessed via Zenodo at https://doi.org/10.5281/zenodo.14896484 ^23^.

## References

[1] US Department of Health and Human Services, Office of Disease Prevention and Health Promotion: Healthy people 2030.Accessed January 15, 2022. Reference Source

[2] Braveman P : What are health disparities and health equity? We need to be clear. *Public Health Rep.* 2014;129 Suppl 2(Suppl 2):5–8. 10.1177/00333549141291S203 24385658 PMC3863701

[3] Frenk J Chen L Bhutta ZA : Health professionals for a new century: transforming education to strengthen health systems in an interdependent world. *Lancet.* 2010;376(9756):1923–1958. 10.1016/S0140-6736(10)61854-5 21112623

[4] Committee on Educating Health Professionals to Address the Social Determinants of Health, Board on Global Health, Institute of Medicine; National Academies of Sciences, Engineering, and Medicine: A framework for educating health professionals to address the social determinants of health.Washington (DC): National Academies Press (US), October 14,2016. 10.17226/21923 27854400

[5] Batra S Orban J Guterbock TM : Social mission metrics: developing a survey to guide health professions schools. *Acad Med.* 2020;95(12):1811–1816. 10.1097/ACM.0000000000003324 32217852 PMC7678650

[6] Farmer N Powell-Wiley TM Middleton KR : Use of a focus group-based cognitive interview methodology to validate a cooking behavior survey among African-American adults. *Front Nutr.* 2022;9: 1000258. 10.3389/fnut.2022.1000258 36545469 PMC9760831

[7] Ouimet JA Bunnage JB Carini RM : Using focus groups to establish the validity and reliability of a college student survey. *Research in Higher Education.* 2024;45:233–50. Reference Source

[8] Liaison Committee on Medical Education document of accreditation standards for medical education programs with full accreditation surveys in the 2022-23 academic year.Published October,2021.

[9] Weiner S : Medical schools overhaul curricula to fight inequities. *AAMC.* May 25,2021. Reference Source

[10] Veras M Pottie K Welch V : Reliability and validity of a new survey to assess global health competencies of health professionals. *Glob J Health Sci.* 2013;5(1):13–27. 10.5539/gjhs.v5n1p13 23283032 PMC4776957

[11] Dupras DM Wieland ML Halvorsen AJ : Assessment of training in health disparities in US internal medicine residency programs. *JAMA Netw Open.* 2020;3(8): e2012757. 10.1001/jamanetworkopen.2020.12757 32777061 PMC7417967

[12] Reutter L Kushner KE : 'Health equity through action on the social determinants of health': taking up the challenge in nursing. *Nurs Inq.* 2010;17(3):269–280. 10.1111/j.1440-1800.2010.00500.x 20712665

[13] Peterson A Charles V Yeung D : The Health Equity Framework: a science- and justice-based model for public health researchers and practitioners. *Health Promot Pract.* 2021;22(6):741–746. 10.1177/1524839920950730 32814445 PMC8564233

[14] Public Health Foundation: Core public health competencies.Public Health Foundation, Updated March 16,2021; Accessed December 12, 2024. Reference Source

[15] Wang YW Davidson MM Yakushko OF : The scale of ethnocultural empathy: development, validation, and reliability. *J Couns Psychol.* 2003;50(2):221–234. 10.1037/0022-0167.50.2.221

[16] Davis MH : A multidimensional approach to individual differences in empathy. *JSAS Catalog of Selected Documents in Psychology.* 1980;10:85. Reference Source

[17] Streiner DL Norman GR Cairney J : Health measurement scales: a practical guide to their development and use.Oxford University Press,2005.

[18] Kane MT : Validating the interpretations and uses of test scores. *J Educ Meas.* 2013;50(1):1–73. 10.1111/jedm.12000

[19] Hoyer D Dee E O'Leary MS : How do we define and measure health equity? The state of current practice and tools to advance health equity. *J Public Health Manag Pract.* 2022;28(5):570–577. 10.1097/PHH.0000000000001603 35867507 PMC9311469

[20] Cortina JM : What is coefficient alpha? An examination of theory and applications. *J Appl Psychol.* 1993;78(1):98–104. 10.1111/jedm.12000

[21] Ward MC Ganjoo R : When I say … health equity. *Med Educ.* 2023;57(2):121–122. 10.1111/medu.14962 36286241 PMC10092194

[22] Mallinson T Stelmack J Velozo C : A comparison of the separation ratio and coefficient alpha in the creation of minimum item sets. *Med Care.* 2004;42(1 Suppl):I17–I24. 10.1097/01.mlr.0000103522.78233.c3 14707752

[23] Ward MC : Student health equity survey running total of survey data. *Zenoda.* 2025. 10.5281/zenodo.14896484

