# Supplementary material for: Initial Validity Evidence for the Student Health Equity Survey: Assessing Knowledge, Attitudes, and Capacity across Health Professions Programs
Source: MedEdPublish (2016). 2025 Nov 7;15:246. [Version 1] doi: 10.12688/mep.21128.1 (PMC13054251; doi:10.12688/mep.21128.1)
Supplement: Peer review history

# Reviewer report: Reviewer response for version 1

**DOI:** 10.21956/mep.22632.r45601

**Published:** 2026-04-06

**Authors:** Ingrid Bahner

**Affiliations:** 1 University of South Florida Morsani College of Medicine, Tampa, Florida, USA

This manuscripts describes the development of an instrument that aims to measure learners’ health equity competencies.

 This instrument fills an important gap in that there are few best practices when designing pathway and bridges programs or health equity curricula. Using a validated instrument would allow to measure the effectiveness of such programs or curricula and would contribute to the much needed development of best practices in this field.

 Using established methods, the authors describe clearly and in great detail the rational design and thorough development process of this instrument. Importantly, they include a discussion on the definition of health equity chosen for the development of this instrument. Limitations are clearly outlined, thus, the authors provide an instrument that others can build upon and has a high potential to advance the field. In addition, the manuscript may serve as a valuable resource for readers who want to develop any kind of psychometric evaluation instrument.

Have any limitations of the research been acknowledged?

Yes

Is the work clearly and accurately presented and does it cite the current literature?

Yes

If applicable, is the statistical analysis and its interpretation appropriate?

Yes

Are all the source data underlying the results available to ensure full reproducibility?

Yes

Is the study design appropriate and is the work technically sound?

Yes

Are the conclusions drawn adequately supported by the results?

Yes

Are sufficient details of methods and analysis provided to allow replication by others?

Yes

Reviewer Expertise:

Mentorship in medical student research

I confirm that I have read this submission and believe that I have an appropriate level of expertise to confirm that it is of an acceptable scientific standard.

# Reviewer report: Reviewer response for version 1

**DOI:** 10.21956/mep.22632.r45090

**Published:** 2026-02-06

**Authors:** Tayseer M Mansour

**Affiliations:** 1 Taibah University, Al Madinah, Saudi Arabia

This manuscript by Ward et al. addresses a critical need in health professions education: the development of a standardized, competency-based instrument to measure health equity knowledge, attitudes, and capacity. The creation of the Student Health Equity Survey (SHES) is a commendable and timely effort. The paper is well-structured, providing a clear and detailed account of the multi-stage process used to gather initial validity evidence, including the use of an expert panel, cognitive interviews, and Rasch analysis. The theoretical grounding in the Health Equity Framework is a significant strength.

 However, while the study's aim and methodological approach are sound, the execution of the pilot study suffers from significant limitations that temper the conclusions. The most critical issue, as also noted by the first reviewer, is a fundamental mismatch between the intended population (incoming, cross-disciplinary health professions students) and the actual sample used for the pilot analysis (predominantly advanced, second year-and-beyond students from a single program). This sampling bias significantly impacts the interpretation of the psychometric data and the generalizability of the findings. Therefore, while the paper has clear academic merit, substantial revisions are required to align the conclusions more closely with the evidence presented.

Have any limitations of the research been acknowledged?

Yes

Is the work clearly and accurately presented and does it cite the current literature?

Yes

If applicable, is the statistical analysis and its interpretation appropriate?

Partly

Are all the source data underlying the results available to ensure full reproducibility?

Yes

Is the study design appropriate and is the work technically sound?

Partly

Are the conclusions drawn adequately supported by the results?

No

Are sufficient details of methods and analysis provided to allow replication by others?

Yes

Reviewer Expertise:

I am an associate professor of medical education. working as program coordinator for MBBcH program. Also, the Head of program assessment committee and faculty development unit.

I confirm that I have read this submission and believe that I have an appropriate level of expertise to confirm that it is of an acceptable scientific standard, however I have significant reservations, as outlined above.

# Reviewer report: Reviewer response for version 1

**DOI:** 10.21956/mep.22632.r44620

**Published:** 2025-11-25

**Authors:** Asmaa Abdelnasser

**Affiliations:** 1 Ibn Sina National College for Medical Studies, Jeddah, Saudi Arabia

They report psychometric findings, refinement iterations, and limitations. The article concludes that SHES provides initial validity evidence and has potential to support curriculum reform in health professions education.

 The manuscript is clear, relevant, and addresses an important gap in health equity assessment. However, several methodological weaknesses, especially sample bias, psychometric issues, and overextended conclusions—need to be addressed before the article can be considered scientifically sound.

**Evaluation According to the Journal Criteria**

**1. Is the work clearly and accurately presented, and does it cite the current literature?**

**Answer: YES**

 The article is well written, logically organized, and uses extensive current literature on health equity, psychometrics, and curriculum development. The conceptual rationale is clear and well grounded.

**No major concerns here.**

**2. Is the study design appropriate and is the work technically sound?**

**Answer: PARTLY**

 The overall design is appropriate for **initial instrument validation**, and the authors correctly use expert review, cognitive interviewing, and Rasch analysis. However, several issues reduce technical soundness:

**Key Problems**

1. **Sampling Bias (Major Issue)**
  - 70% of participants were **not first-year students**, even though the SHES is intended for *incoming* students.
  - 79% of participants were from **one program (Biomedical Laboratory Sciences)**, limiting cross-disciplinary validity.
2. **Knowledge Subscale Reliability Extremely Low (α = 0.46)**
  The authors attribute this to ceiling effects, but this needs clearer justification and further analysis.
3. **Rasch Dimensionality Concerns**
  Several items showed misfit: some subscales initially failed uni-dimensionality tests.

**Recommendations to Address**

**MUST ADDRESS for scientific soundness**

- Provide a clearer justification for using a non-novice sample.
- Discuss more explicitly how sample characteristics may have biased psychometric outcomes.
- Add detail about how these limitations will be corrected in future validation phases.
- Offer a plan for validating the SHES specifically with incoming students.

**SHOULD ADDRESS**

- Describe whether misfitting items will be revised or removed in future work.
- Explain why Rasch was chosen over other models and whether alternative analyses (e.g., CFA) are planned.

**3. Are sufficient details of methods and analysis provided to allow replication?**

**Answer: YES**

 The manuscript includes enough detail to replicate the study:

- Exact survey items (Table 2).
- Subscale structure and scoring explanations.
- Rasch software version, thresholds, and criteria.
- Demographic table (Table 3).
- Data availability through Zenodo.

**Minor Improvement Suggestion**

 Provide the focus group interview guide as an appendix to support full transparency.

**4. If applicable, is the statistical analysis and its interpretation appropriate?**

**Answer: PARTLY**

**Strengths**

- Rasch modeling is an appropriate choice for early assessment of internal structure.
- Cronbach’s alpha and correlation analyses are correctly used.
- Item-level misfit statistics are transparently reported.

**Concerns**

1. **Interpretation of Low Knowledge Subscale Reliability**
  α = 0.46 suggests poor internal consistency, not only ceiling effects.
  More analysis (item-total correlations, but response distributions also is needed.
2. **Very High Alpha (0.97) for One Subscale**
  Likely indicates redundancy among items.
3. **Multidimensionality in Rasch PCA**
  The authors describe collapsing subscales but provide insufficient detail on the decision process.
4. **Social Desirability Bias Not Accounted For**
  Attitudinal items regarding racism and equity are highly susceptible to this.

**Recommendations to Improve Statistical Rigor**

**MUST ADDRESS**

- Add a more thorough interpretation of subscale reliabilities.
- Clarify decision-making criteria for collapsing subscales into four categories.

**SHOULD ADDRESS**

- Discuss how future work will evaluate **predictive validity**, **test–retest reliability**, and **social desirability impacts**.

**5. Have any limitations of the research been acknowledged?**

**Answer: YES**

 This is handled well.

 The manuscript includes a well-developed limitations section addressing:

- Sample bias
- Rasch sensitivity
- COVID-19 disruptions
- Issues with reliability and potential item redundancy
- Generalizability concerns

**6. Are all the source data underlying the results available to ensure full reproducibility?**

**Answer: YES**

**7. Are the conclusions drawn adequately supported by the results?**

**Answer: PARTLY**

**Well-Supported Conclusions**

- The SHES demonstrates **initial validity evidence**
- SHES may help identify curriculum needs and gaps.
- SHES appears to measure four distinct constructs.

**Overextended Conclusions**

1. **Claim of Cross-Disciplinary Applicability**
  Not fully supported due to overrepresentation of a single program.
2. **Use for Incoming First-Year Students**
  The sample was primarily made of advanced students.
3. **Implications for Real-World Competence**
  No behavioral outcomes were studied.

**Recommendations**

**MUST ADDRESS**

- Modify conclusions to align strictly with the available evidence.
- Remove or revise claims about generalizability across programs.
- Avoid implying predictive validity for clinical or professional performance.

**SHOULD ADDRESS**

- Indicate clearly that further validation is required before high-stakes use.

**Overall Recommendation**

**Decision: Minor to Moderate Revision Needed**

 The manuscript is promising and relevant, but to be scientifically sound, the authors must:

**Issues That MUST Be Addressed**

1. **Sampling bias and implications for psychometric findings**
2. **Clearer justification and interpretation of subscale reliability**
3. **More cautious and accurate conclusions, especially regarding generalizability**
4. **Clarify Rasch dimensionality decisions and misfit item implications**

**Issues That Should Be Addressed**

- Provide more details about future validation steps
- Consider discussing social desirability bias
- Improve clarity on item redundancy

They report psychometric findings, refinement iterations, and limitations. The article concludes that SHES provides initial validity evidence and has potential to support curriculum reform in health professions education.

 The manuscript is clear, relevant, and addresses an important gap in health equity assessment. However, several methodological weaknesses, especially sample bias, psychometric issues, and overextended conclusions—need to be addressed before the article can be considered scientifically sound.

**Evaluation According to the Journal Criteria**

**1. Is the work clearly and accurately presented, and does it cite the current literature?**

**Answer: YES**

 The article is well written, logically organized, and uses extensive current literature on health equity, psychometrics, and curriculum development. The conceptual rationale is clear and well grounded.

**No major concerns here.**

**2. Is the study design appropriate and is the work technically sound?**

**Answer: PARTLY**

 The overall design is appropriate for **initial instrument validation**, and the authors correctly use expert review, cognitive interviewing, and Rasch analysis. However, several issues reduce technical soundness:

**Key Problems**

1. **Sampling Bias (Major Issue)**
  - 70% of participants were **not first-year students**, even though the SHES is intended for *incoming* students.
  - 79% of participants were from **one program (Biomedical Laboratory Sciences)**, limiting cross-disciplinary validity.
2. **Knowledge Subscale Reliability Extremely Low (α = 0.46)**
  The authors attribute this to ceiling effects, but this needs clearer justification and further analysis.
3. **Rasch Dimensionality Concerns**
  Several items showed misfit: some subscales initially failed uni-dimensionality tests.

**Recommendations to Address**

**MUST ADDRESS for scientific soundness**

- Provide a clearer justification for using a non-novice sample.
- Discuss more explicitly how sample characteristics may have biased psychometric outcomes.
- Add detail about how these limitations will be corrected in future validation phases.
- Offer a plan for validating the SHES specifically with incoming students.

**SHOULD ADDRESS**

- Describe whether misfitting items will be revised or removed in future work.
- Explain why Rasch was chosen over other models and whether alternative analyses (e.g., CFA) are planned.

**3. Are sufficient details of methods and analysis provided to allow replication?**

**Answer: YES**

 The manuscript includes enough detail to replicate the study:

- Exact survey items (Table 2).
- Subscale structure and scoring explanations.
- Rasch software version, thresholds, and criteria.
- Demographic table (Table 3).
- Data availability through Zenodo.

**Minor Improvement Suggestion**

 Provide the focus group interview guide as an appendix to support full transparency.

**4. If applicable, is the statistical analysis and its interpretation appropriate?**

**Answer: PARTLY**

**Strengths**

- Rasch modeling is an appropriate choice for early assessment of internal structure.
- Cronbach’s alpha and correlation analyses are correctly used.
- Item-level misfit statistics are transparently reported.

**Concerns**

1. **Interpretation of Low Knowledge Subscale Reliability**
  α = 0.46 suggests poor internal consistency, not only ceiling effects.
  More analysis (item-total correlations, but response distributions also is needed.
2. **Very High Alpha (0.97) for One Subscale**
  Likely indicates redundancy among items.
3. **Multidimensionality in Rasch PCA**
  The authors describe collapsing subscales but provide insufficient detail on the decision process.
4. **Social Desirability Bias Not Accounted For**
  Attitudinal items regarding racism and equity are highly susceptible to this.

**Recommendations to Improve Statistical Rigor**

**MUST ADDRESS**

- Add a more thorough interpretation of subscale reliabilities.
- Clarify decision-making criteria for collapsing subscales into four categories.

**SHOULD ADDRESS**

- Discuss how future work will evaluate **predictive validity**, **test–retest reliability**, and **social desirability impacts**.

**5. Have any limitations of the research been acknowledged?**

**Answer: YES**

 This is handled well.

 The manuscript includes a well-developed limitations section addressing:

- Sample bias
- Rasch sensitivity
- COVID-19 disruptions
- Issues with reliability and potential item redundancy
- Generalizability concerns

**6. Are all the source data underlying the results available to ensure full reproducibility?**

**Answer: YES**

**7. Are the conclusions drawn adequately supported by the results?**

**Answer: PARTLY**

**Well-Supported Conclusions**

- The SHES demonstrates **initial validity evidence**
- SHES may help identify curriculum needs and gaps.
- SHES appears to measure four distinct constructs.

**Overextended Conclusions**

1. **Claim of Cross-Disciplinary Applicability**
  Not fully supported due to overrepresentation of a single program.
2. **Use for Incoming First-Year Students**
  The sample was primarily made of advanced students.
3. **Implications for Real-World Competence**
  No behavioral outcomes were studied.

**Recommendations**

**MUST ADDRESS**

- Modify conclusions to align strictly with the available evidence.
- Remove or revise claims about generalizability across programs.
- Avoid implying predictive validity for clinical or professional performance.

**SHOULD ADDRESS**

- Indicate clearly that further validation is required before high-stakes use.

**Overall Recommendation**

**Decision: Minor to Moderate Revision Needed**

 The manuscript is promising and relevant, but to be scientifically sound, the authors must:

**Issues That MUST Be Addressed**

1. **Sampling bias and implications for psychometric findings**
2. **Clearer justification and interpretation of subscale reliability**
3. **More cautious and accurate conclusions, especially regarding generalizability**
4. **Clarify Rasch dimensionality decisions and misfit item implications**

**Issues That Should Be Addressed**

- Provide more details about future validation steps
- Consider discussing social desirability bias
- Improve clarity on item redundancy

Have any limitations of the research been acknowledged?

Yes

Is the work clearly and accurately presented and does it cite the current literature?

Yes

If applicable, is the statistical analysis and its interpretation appropriate?

Partly

Are all the source data underlying the results available to ensure full reproducibility?

Yes

Is the study design appropriate and is the work technically sound?

Partly

Are the conclusions drawn adequately supported by the results?

Partly

Are sufficient details of methods and analysis provided to allow replication by others?

Yes

Reviewer Expertise:

Health Professions Education

I confirm that I have read this submission and believe that I have an appropriate level of expertise to confirm that it is of an acceptable scientific standard, however I have significant reservations, as outlined above.
